# Iatrogenic *Mycobacterium simiae* Skin Infection in an Immunocompetent Patient

**DOI:** 10.3201/eid1005.030681

**Published:** 2004-05

**Authors:** Jaime Piquero, Vanesa Piquero Casals, Edgar Larotta Higuera, Mitchell Yakrus, David Sikes, Jacobus H. de Waard

**Affiliations:** * Departamento de Dermatología, Clínica Sanatrix, Caracas, Venezuela; † Centers for Disease Control and Prevention, Atlanta, Georgia, USA; ‡ Laboratorio de Tuberculosis, Instituto de Biomedicina, ,Caracas, Venezuela

**Keywords:** Skin infection, mesotherapy, intradermal injection, *Mycobacterium simiae*, nontuberculous mycobacteria

**To the Editor**: We report a case of a 36-year-old woman who sought treatment for 45 firm and erythematous nodular lesions on her face and neck. A physical examination showed no other abnormalities. Results of a chest x-ray and routine laboratory tests were normal. The patient tested negative for hepatitis B and HIV. Three weeks before she sought treatment, the patient reported receiving multiple intradermal microinjections in her face and neck for cosmetic purposes (mesotherapy) with an unlicensed product consisting of a solution of glycosaminoglycans. The injections had been administered by an unlicensed practitioner in a nonmedical office setting. The patient stated that 2 days after the therapy, a fever developed; it persisted for several days, along with redness at the inoculation sites, which gradually developed into nodules.

Standard staining of a biopsied specimen from the lesion site was negative for bacteria, fungi, and mycobacteria. A histopathologic examination of a biopsy specimen showed an unspecific granulomatous infiltrate. Culture for common bacteria and fungi was negative, but culture of a sterile nodule aspirate on Lowenstein-Jensen medium was positive for acid-fast bacteria after 5 weeks. By using restriction endonuclease analysis of the 65-kDa heat shock protein gene (*1*), we found that the isolate showed a pattern compatible with *Mycobacterium simiae.* Identification was subsequently confirmed by high performance liquid chromatography of mycolic acids at the Centers for Disease Control and Prevention, Atlanta, Georgia. The isolate was tested for drug susceptibility against a panel of drugs and found to be resistant to most drugs tested (streptomycin, isoniazid, rifampin, ethambutol, ethionamide, rifabutin, ciprofloxacin, kanamycin, capreomycin, p-aminosalicylic acid, ofloxacin, and amikacin) and susceptible to clarithromycin at an MIC of 1 μg/mL. Treatment with clarithromycin was started, and the granulomas slowly cleared after 9 months of treatment.

To our knowledge, this is the first reported case of an iatrogenic skin infection caused by *M. simiae* in an immunocompetent person. *M. simiae* is a species of nontuberculous mycobacterium commonly found in nature, but its role as a pathogen has been controversial. The slow-growing, photochromogenic mycobacterium has been isolated from both surface and tap water and has been associated with a nosocomial pseudo-outbreak suspected to have originated from a contaminated hospital water supply (*2*). *M.* *simiae* rarely causes disease in immunocompetent patients; most infections are associated with AIDS patients (*3*–*5*).

Although this patient responded to treatment with clarithromycin, no established optimal therapeutic regimen exists against this species of *Mycobacterium*. *M. simiae* is often multidrug resistant, but successful therapy with clarithromycin in combination with ethambutol and ciprofloxacin has been reported in AIDS patients (*6*,*7*).

We conclude that *M. simiae* can cause skin infections if injected directly into the dermis. Prolonged treatment is necessary to cure the patient of the infection. This report underscores the risk from alternative therapies performed with unlicensed products and by unlicensed practitioners. Unusual infectious agents should be considered when diagnosing skin infection in patients who have received injections for cosmetic purposes.
